# Effect of sinus rhythm restoration on markers of thrombin generation in atrial fibrillation

**DOI:** 10.1186/s12959-017-0153-1

**Published:** 2017-12-28

**Authors:** Anja Wiedswang Horjen, Ingebjørg Seljeflot, Trygve Berge, Pål Smith, Harald Arnesen, Arnljot Tveit

**Affiliations:** 1 Department of Medical Research, Bærum Hospital, Vestre Viken Hospital Trust, N-3004 Drammen, Norway; 2 Faculty of Medicine, University of Oslo, Oslo, Norway; 3 Center for Clinical Heart Research, Department of Cardiology, Oslo University Hospital Ullevål, Oslo, Norway; 4 Department of Cardiology, Akershus University Hospital, Lørenskog, Norway

**Keywords:** Atrial fibrillation, Cardioversion, Hypercoagulability, Thrombin generation

## Abstract

**Background:**

Atrial fibrillation (AF) confers a hypercoagulable state; however, it is not clear whether restoration of sinus rhythm is associated with normalisation of markers of thrombogenesis. We studied the impact of sustained sinus rhythm on prothrombotic markers, and their predictive abilities in foreseeing rhythm outcome after cardioversion.

**Methods:**

In a double blind, placebo-controlled study, 171 patients referred for electrical cardioversion of persistent AF were randomised to receive candesartan or placebo for 3-6 weeks before and 6 months after cardioversion. Endogenous thrombin potential (ETP), prothrombin fragment 1 + 2 (F1 + 2) and D-dimer were measured before cardioversion and at end of study. These markers were also measured in a reference group comprising 49 subjects without AF.

**Results:**

The markers remained unchanged in those 28 patients who maintained sinus rhythm. Discontinuation of warfarin treatment in a subset of 13 low-risk patients in sinus rhythm was associated with significantly higher levels of D-dimer and F1 + 2 compared to the reference group; D-dimer (456 ng/mL (276, 763) vs. 279 ng/mL (192, 348), *p* = 0.002) and F1 + 2 (700 pmol/L (345, 845) vs. 232 pmol/L (190, 281), *p* < 0.001). None of the markers were associated with rhythm outcome after electrical cardioversion.

**Conclusions:**

Sustained sinus rhythm for 6 months after cardioversion for AF had no impact on ETP, F1 + 2 or D-dimer levels. Discontinuation of warfarin in low-risk patients with sustained sinus rhythm was associated with significantly higher levels of D-dimer and F1 + 2 compared to the reference group. Our results suggest persistent hypercoagulability in AF patients despite long-term maintenance of sinus rhythm.

**Trial registration:**

The CAPRAF study was registered at clinicaltrials.gov (NCT00130975) in August 2005.

## Background

Atrial fibrillation (AF) is a major risk factor for thromboembolic events [1]. The hypercoagulability in AF is related to blood stasis, endocardial changes and abnormal blood constituents including increased markers of thrombogenesis [2–4]. The prothrombotic state in AF is adversely affected by electrical cardioversion [5, 6], and current guidelines recommend that anticoagulation should be continued lifelong in patients with risk factors of stroke or AF recurrence, irrespective of apparent maintenance of sinus rhythm following cardioversion [7]. Whether the activation of the coagulation system persists or is attenuated after prolonged periods of sinus rhythm, remains an open question.

Haemostatic alterations as a consequence of AF are widely accepted. Intriguingly, it has recently been suggested that hypercoagulability in itself causes atrial fibrosis and thereby promotes a substrate for AF [8]. AF is associated with elevated levels of prothrombin fragment 1 + 2 (F1 + 2) [9]. F1 + 2 is released during the conversion of prothrombin to thrombin, whereas the endogenous thrombin potential (ETP) indicates an ex vivo potential for thrombin generation. Measurement of D-dimer reflects both thrombin generation and fibrin turnover, and may complement clinical and echocardiographic risk stratification for stroke and thromboembolism in AF [10–12]. The predictive abilities of ETP, F1 + 2 and D-dimer in foreseeing rhythm outcome after electrical cardioversion for AF have not previously been reported.

The objectives of the present investigation were twofold. First, to study the effects of sustained sinus rhythm after electrical cardioversion on levels of ETP, F1 + 2 and D-dimer, and to compare these markers in low-risk AF patients in sinus rhythm with levels measured in a reference group without AF. Secondly, we evaluated the prognostic abilities of ETP, F1 + 2 and D-dimer in foreseeing rhythm outcome 6 months after electrical cardioversion.

## Methods

### Study design

**Fig. 1:**
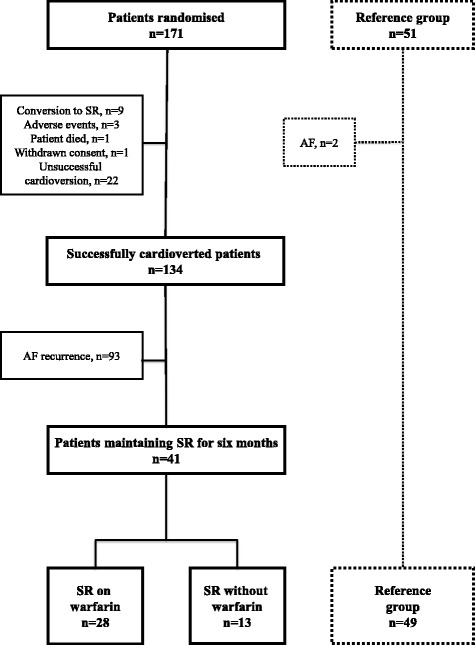
Flow chart of the CAPRAF study and reference group recruited from the ACE 1950 study. Abbreviations: ACE, Akershus Cardiac Examination 1950 study; AF, atrial fibrillation; CAPRAF study, Candesartan in the Prevention of Relapsing Atrial Fibrillation study; n, number of patients; SR, sinus rhythm

The present study is a substudy of the double blind, placebo-controlled Candesartan in the Prevention of Relapsing Atrial Fibrillation (CAPRAF) study [13]. Briefly, 171 patients with AF were randomised to receive candesartan 8 mg once daily (*n* = 86) or placebo (*n* = 85) for 3 to 6 weeks before and then candesartan 16 mg once daily or placebo for 6 months after electrical cardioversion (Fig. 1). Patients with congestive heart failure or renal impairment were not included in the study. Cardioversion was deemed successful if sinus rhythm was established and maintained for at least 2 h (*n* = 134). Relapse of AF was defined as first electrocardiogram–recorded episode of AF. Blood samples were collected at baseline and at 6 months’ follow-up. CHA_2_DS_2_-VASc score was used to stratify patients according to risk of stroke, with scores ranging from 0 to 9 and higher scores indicating greater risk. The scoring system assigns one point for each of the following; congestive heart failure, hypertension, age 65-74 years, diabetes, vascular disease or female sex, whereas age ≥ 75 years and previous stroke or transient ischemic attack count two points each. The study was approved by the Regional Ethics Committee, and all patients provided written, informed consent in accordance with the revised Declaration of Helsinki. The CAPRAF study is registered at clinicaltrials.gov (NCT00130975).

Residents of Asker and Bærum municipalities participating in a pilot for the Akershus Cardiac Examination (ACE) 1950 study were included as reference group (*n* = 49) [14]. Fifty-one subjects accepted the invitation to this pilot. Two subjects were diagnosed with AF, and therefore excluded from the reference group (Fig. 1). Approval was obtained by the Regional Ethics Committee, and all patients provided written, informed consent in accordance with the revised Declaration of Helsinki. The ACE 1950 study is registered at clinicaltrials.gov (NCT01555411).

### Laboratory analyses

After centrifugation, citrated plasma was aliquoted and kept frozen at −70 °C until analysed. Levels of F1 + 2 and D-dimer were assessed by commercially available enzyme immunoassays; Enzygnost® F1 + 2 (monoclonal) (Siemens, Marburg, Germany) and Asserachrom® D-dimer (Stago Diagnostica). The coefficients of variation were 5.4% for F1 + 2 and 6.5% for D-dimer.

Ex-vivo thrombin generation potential was investigated by the calibrated automated thrombogram (CAT) assay, performed according to the manufacturer’s instruction (Thrombinoscope BV, Maastricht, The Netherlands). The method is described in detail elsewhere [15]. Briefly, platelet poor plasma was mixed with a reagent containing relipidated tissue factor and phospholipids, with the final concentrations of 5 pM and 4 μM, respectively. Reagents were provided from Thrombinoscope BV (Maastricht, The Netherlands). The reactions were performed in micro titer wells after automatic addition of a fresh made starting reagent containing CaCl_2_ (100 mM) and a thrombin specific fluorogenic substrate (Z-Gly-Gly-Arg-AMC) (2.5 mM). The fluorescence intensity was recorded by the Fluoroskan Ascent® micro plate fluorometer (Thermo Fisher Scientific Oy, Vantaa, Finland). By simultaneous analysis of an inert thrombin calibrator with known thrombin activity, the software program (Thrombinoscope BV, version 3.0.0.29) is enabled to display the ETP (nM*min). The coefficient of variation was 5.9% for ETP.

### Statistical analyses

Data are presented as mean ± standard deviation for normally distributed variables, while continuous variables not normally distributed are expressed as median (25^th^, 75^th^ percentiles). Categorical variables are shown as frequencies (%). Continuous variables were analysed by Student *t* test or the Mann-Whitney *U*-test depending on distribution. Categorical data were compared by the Chi-square test or Fischer’s exact test where appropriate. The impact of continuous clinical variables and CHA_2_DS_2_-VASc score on haemostatic markers was analysed using bivariate non-parametric correlations (Spearman, correlation coefficient denoted r_s_). Kaplan-Meier curves for the probability of first recurrence of AF were plotted for medians and quartiles of baseline levels of the haemostatic markers and compared by log-rank test. Group comparisons were assessed by Mann-Whitney *U*-test. Kruskal-Wallis H test was used to compare levels of the markers according to CHA_2_DS_2_-VASc risk groups. For pairwise comparisons Bonferroni adjusted Mann-Whitney *U*-test was used. Wilcoxon’s matched-pairs test was used to compare baseline and end-of-study levels of the markers. The effects of treatment with candesartan and discontinued warfarin treatment on prothrombotic marker levels were assessed with ANCOVA regression analysis on logarithmically transformed data. To investigate the impact of potential confounders on the relation between AF and the prothrombotic markers, variables related to both AF and the haemostatic indices with a *p*-value of < 0.20 were included in a multivariate linear regression model, with logarithmically transformed marker values as the dependent variable. Medications were not included in the multivariate analysis because they were thought only to reflect the diseases that indicated their use. A two-sided *p*-value of < 0.05 was considered statistically significant. Statistical analyses were performed with IBM SPSS Statistics for Windows, version 23.0 (IBM Corp., New York, USA).

## Results

Baseline levels of the prothrombotic markers were available in 134 (33 women/101 men) successfully cardioverted patients (Table 1). The mean age was 64 ± 11 years (range 21-84). The mean CHA_2_DS_2_-VASc score of this population was 1.5 ± 1.3 (range 0-5). The prothrombin time, expressed as the International Normalised Ratio (INR), was 2.3 ± 0.7 at baseline.

**Table 1:** Characteristics of the successfully cardioverted atrial fibrillation patients

Variable	*n* = 134
*Medical history*	
Age (years)	64 ± 11
Sex (women/men)	33/101
Body mass index (kg/m^2^)	26 ± 4
Hypertension	38 (28%)
Coronary heart disease	12 (9%)
Diabetes	10 (7%)
Chronic obstructive pulmonary disease	7 (5%)
Current cigarette smoking	21 (16%)
CHA_2_DS_2_-VASc score	1.5 ± 1.3
*Medication at randomisation*	
Angiotensin receptor blocker/study drug	66 (49%)
Digitalis	16 (12%)
Beta-blockers	48 (36%)
Calcium channel blockers	63 (47%)
Diuretics	11 (8%)
Statins	18 (13%)
*Blood pressure and heart rate*	
Systolic blood pressure (mmHg)	134 ± 18
Diastolic blood pressure (mmHg)	82 ± 8
Ventricular heart rate (beats per minute)	85 ± 17
*Echocardiographic assessments*	
Left atrial systolic diameter (mm)	46 ± 6
Left atrial systolic area (cm^2^)	27 ± 5
Fraction of shortening (%)	30 ± 7

Baseline clinical characteristics of the 134 successfully cardioverted patients in the Candesartan in the Prevention of Relapsing Atrial Fibrillation (CAPRAF) study. Values presented as mean ± standard deviation or number (%). *Abbreviations*: *CHA*_*2*_ *DS*_*2*_ *-VASc score* A measure of stroke risk in patients with atrial fibrillation, with scores ranging from 0 to 9 and higher scores indicating greater risk, *n* Number of patients

Median baseline levels of D-dimer (357 ng/mL (226, 524)) and F1 + 2 (190 pmol/L (140, 310)) have been reported previously [16]. Median baseline level of ETP was 474 nM*min (366, 620). A correlation was seen between F1 + 2 and ETP (r_s_ = 0.594, *p* < 0.001). D-dimer correlated to F1 + 2 (r_s_ = 0.479, *p* < 0.001), but not to ETP (r_s_ = 0.151, *p* = 0.087).

D-dimer correlated to age (r_s_ = 0.331, *p* < 0.001), left atrial area (r_s_ = 0.182, *p* = 0.040) and left atrial diameter (r_s_ = 0.201, *p* = 0.022) [16]. Subjects with diabetes had higher levels of D-dimer; (491 ng/mL (290, 671) vs. 350 ng/mL (219, 500), *p* = 0.045). Levels of F1 + 2 correlated to left atrial area (r_s_ = 0.183, *p* = 0.039) and left atrial diameter (r_s_ = 0.193, *p* = 0.028). Levels of ETP correlated to left atrial area (r_s_ = 0.181, *p* = 0.042) and to body mass index (r_s_ = 0.194, *p* = 0.032). Females had lower levels of ETP as compared to males; (436 nM*min (292, 526) vs. 493 nM*min (391, 623), *p* = 0.035). CHA_2_DS_2_-VASc score correlated weakly, but significantly with baseline levels of D-dimer (r_s_ = 0.213, *p* = 0.015). An inverse correlation was observed between CHA_2_DS_2_-VASc score and baseline levels of ETP (r_s_ = −0.215, *p* = 0.014). Baseline levels of ETP were significantly higher in patients with low CHA_2_DS_2_-VASc score (0-1) as compared to patients with CHA_2_DS_2_-VASc score ≥ 3; (534 nM*min (419, 705) vs. 417 nM*min (276, 539), *p* = 0.011).

**Fig. 2:**
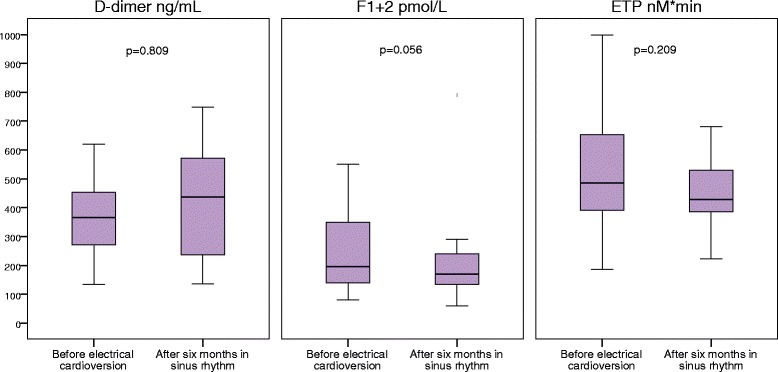
Marker levels in patients with sustained SR and continued warfarin treatment. *P*-values derived from the Wilcoxon signed-rank test. Center lines show the medians; box limits indicate the 25th and 75th percentiles; whiskers extend 1.5 times the interquartile range from the 25^th^ and 75^th^ percentiles. Abbreviations: ETP, endogenous thrombin potential; F1 + 2, prothrombin fragment 1 + 2; SR, sinus rhythm

In order to evaluate the potential impact of sustained sinus rhythm on levels of prothrombotic markers, baseline blood samples drawn before electrical cardioversion were compared with levels at study end in patients with 6 months’ survival-free of AF and with continued warfarin treatment throughout the study. The prothrombotic markers remained unchanged in the 28 patients with sustained sinus rhythm for 6 months and who were treated with warfarin till study end; D-dimer (367 ng/mL (271, 454) vs. 437 ng/mL (237, 572), *p* = 0.809), F1 + 2 (195 pmol/L (140, 350) vs. 170 pmol/L (135, 240), *p* = 0.056) and ETP (485 nM*min (392, 653) vs. 429 nM*min (362, 531), *p* = 0.209) (Fig. 2). Baseline levels of INR were comparable with study end levels; (2.3 ± 0.8 vs. 2.4 ± 0.5, *p* = 0.914).

Warfarin was discontinued in 13 AF patients after successful cardioversion (Fig. 1). These patients were younger (59 ± 12 years vs. 68 ± 9 years, *p* = 0.008), had a lower prevalence of hypertension (15% vs. 43%, *p* = 0.156) and a lower CHA_2_DS_2_-VASc score (1.2 ± 1.3 vs. 2.1 ± 1.5, *p* = 0.177) compared to those 28 AF patients who continued warfarin treatment (Table 2). The time interval between warfarin discontinuation and study end was 106 ± 29 days (range 45-146). A significant rise in all markers was seen in the 13 patients who discontinued warfarin treatment; D-dimer (456 ng/mL (276, 763) vs. 304 ng/mL (165,470), *p* = 0.002), F1 + 2 (700 pmol/L (345, 845) vs. 160 pmol/L (110, 190), *p* = 0.034) and ETP (1279 nM*min (1069, 1496) vs. 417 nM*min (339, 494), *p* = 0.001). ANCOVA analysis comparing the changes in prothrombotic markers from baseline to study end showed significant effects of discontinued warfarin treatment in the 13 low-risk AF patients; D-dimer (*p* = 0.006), F1 + 2 (*p* < 0.001) and ETP (*p* < 0.001).

**Table 2:** AF patients in SR 6 months after cardioversion according to warfarin treatment at study end

Variable	No warfarin (*n* = 13)	Warfarin (*n* = 28)	*p*
*Medical history*			
Age (years)	59 ± 12	68 ± 9	0.008
Sex (women/men)	4/9	6/22	0.698
Body mass index (kg/m^2^)	25 ± 3	25 ± 2	0.602
Hypertension	2 (15%)	12 (43%)	0.156
Coronary heart disease	1 (8%)	5 (18%)	0.645
Diabetes	1 (8%)	0	0.317
Chronic obstructive pulmonary disease	0	1 (4%)	1.0
Current cigarette smoking	3 (23%)	2 (7%)	0.307
CHA_2_DS_2_-VASc score	1.2 ± 1.3	2.1 ± 1.5	0.177
*Medication*			
Angiotensin receptor blocker/study drug	6 (46%)	12 (43%)	0.843
Angiotensin-converting enzyme inhibitor	0	0	–
Beta-blockers	3 (23%)	11 (39%)	0.481
Calcium channel blockers	3 (23%)	8 (29%)	1.0
Diuretics	0	2 (7%)	1.0
Statins	1 (8%)	9 (32%)	0.129
Antiplatelet therapy	2 (15%)	0	0.095
*Blood pressure and heart rate*			
Systolic blood pressure (mmHg)	129 ± 18	140 ± 17	0.060
Diastolic blood pressure (mmHg)	78 ± 6	80 ± 7	0.401
Ventricular heart rate (beats per minute)	59 ± 7	58 ± 14	0.857

Clinical characteristics of atrial fibrillation patients maintaining sinus rhythm for 6 months after electrical cardioversion with (*n* = 28) and without (*n* = 13) warfarin treatment. Values presented as mean ± standard deviation or number (%). *Abbreviations*: *AF* Atrial fibrillation; CHA_2_DS_2_-VASc score, a measure of stroke risk in patients with atrial fibrillation, with scores ranging from 0 to 9 and higher scores indicating greater risk, *n* Number of patients, *SR* Sinus rhythm

**Fig. 3:**
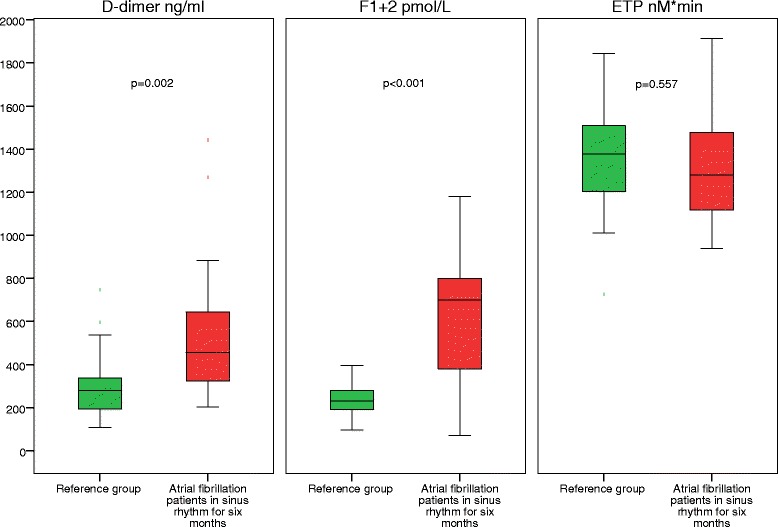
Marker levels in patients with sustained SR and discontinued warfarin treatment compared to reference group. P-values are derived from the Mann-Whitney *U*-test. Center lines show the medians; box limits indicate the 25^th^ and 75^th^ percentiles; whiskers extend 1.5 times the interquartile range from the 25^th^ and 75^th^ percentiles. Abbreviations: ETP, endogenous thrombin potential; F1 + 2, prothrombin fragment 1 + 2; SR, sinus rhythm

Marker values in the 13 patients in sinus rhythm at end of study were compared with corresponding values of 49 subjects in the reference group (Table 3). In these 13 AF patients, D-dimer and F1 + 2 were significantly elevated compared to the reference group; D-dimer (456 ng/mL (276, 763) vs. 279 ng/mL (192, 348), *p* = 0.002) and F1 + 2 (700 pmol/L (345, 845) vs. 232 pmol/L (190, 281), *p* < 0.001) (Fig. 3). There were no significant differences in levels of ETP; (1279 nM*min (1069, 1496) vs. 1377 nM*min (1204, 1509), *p* = 0.557) (Fig. 3). Hypertension was the only variable that met our criteria for potential confounders. In multivariate, linear regression analysis, AF remained the only significant determinant of both markers; D-dimer (0.50 (0.15, 0.43), *p* < 0.001, R^2^ = 0.25) and F1 + 2 (0.60 (0.22, 0.48), *p* < 0.001, R^2^ = 0.36).

**Table 3:** AF patients in SR with discontinued warfarin treatment at study end versus the reference group

Variable	AF patients (*n* = 13)	Reference group (*n* = 49)	*p*
*Medical history*			
Age (years)	59 ± 12	62 ± 0	0.298
Sex (women/men)	4/9	20/29	0.509
Body mass index (kg/m^2^)	25 ± 3	26 ± 4	0.253
Hypertension	2 (15%)	18 (37%)	0.192
Coronary heart disease	1 (8%)	2 (4%)	0.513
Diabetes	1 (8%)	4 (8%)	1.0
Chronic obstructive pulmonary disease	0	1 (2%)	1.0
Current cigarette smoking	3 (23%)	7 (14%)	0.432
*Medication*			
Angiotensin receptor blocker/study drug	6 (46%)	13 (27%)	0.192
Angiotensin-converting enzyme inhibitor	0	1 (2%)	1.0
Beta-blockers	3 (23%)	4 (8%)	0.153
Calcium channel blockers	3 (23%)	4 (8%)	0.153
Diuretics	0	4 (8%)	0.571
Statins	1 (8%)	11 (22%)	0.431
Antiplatelet therapy	2 (15%)	5 (10%)	0.630
*Blood pressure and heart rate*			
Systolic blood pressure (mmHg)	129 ± 18	134 ± 15	0.326
Diastolic blood pressure (mmHg)	78 ± 6	78 ± 8	0.779
Ventricular heart rate (beats per minute)	59 ± 7	64 ± 10	0.072

Clinical characteristics of atrial fibrillation patients maintaining sinus rhythm for 6 months without warfarin treatment at study end (*n* = 13) versus the reference group (*n* = 49).Values presented as mean ± standard deviation or number (%). *Abbreviations*: *AF* Atrial fibrillation; *n* Number of patients, *SR* Sinus rhythm

None of the markers were predictive of rhythm outcome 6 months after electrical cardioversion when dichotomised by median, baseline levels; D-dimer (log rank, *p* = 0.849), ETP (log rank, *p* = 0.423) and F1 + 2 (log rank, *p* = 0.638). Kaplan-Meier analysis of quartiles of all three markers showed similar curves for survival free of AF for each quartile: D-dimer (log rank, *p* = 0.750), ETP (log rank, *p* = 0.346) and F1 + 2 (log rank, *p* = 0.586).

Treatment with candesartan had no impact on the levels of prothrombotic markers. ANCOVA analysis comparing the changes in prothrombotic markers from baseline to study end according to randomisation group showed no significant effects of candesartan (data not shown).

## Discussion

Sustained sinus rhythm for 6 months after electrical cardioversion for AF had no impact on levels of ETP, F1 + 2 or D-dimer. Furthermore, none of the markers were predictive of rhythm outcome after electrical cardioversion. However, discontinuation of warfarin treatment in a subset of 13 low-risk patients in sinus rhythm was associated with significantly higher levels of D-dimer and F1 + 2 compared to the reference group. Thus, AF patients maintain a hypercoagulable state despite restoration and maintenance of sinus rhythm 6 months after successful cardioversion.

Angiotensin II may initiate a prothrombotic state by inducing inflammation, endothelial dysfunction and activation of platelets [17]. Therefore, it is plausible that angiotensin II receptor blockers could influence on hypercoagulability. However, we found no effect of candesartan on levels of haemostatic markers in the present study.

The prothrombotic markers remained unchanged after restoration and maintenance of sinus rhythm for 6 months in patients with continued warfarin treatment throughout the study. Other studies are in line with our findings. Li-Saw-Hee et al. investigated three different markers related to thrombogenicity; namely fibrinogen, P-selectin and von Willebrand factor, and observed no changes in these markers after 3 months maintenance of sinus rhythm following cardioversion [18]. Another study reports unchanged levels of D-dimer, von Willebrand factor and soluble thrombomodulin 1 month after either spontaneous restoration of sinus rhythm or pharmacological cardioversion for acute onset AF without anticoagulation treatment [19]. Hence, sustained sinus rhythm seems to have little impact on the hypercoagulable state associated with AF.

We observed a rise in all three markers of hypercoagulability following discontinued warfarin treatment in 13 low-risk AF patients with sinus rhythm for 6 months. This observation is in line with previous reports of lowered levels of D-dimer, F1 + 2 and ETP by anticoagulation therapy [20–22]. Interestingly, we observed D-dimer and F1 + 2 to be significantly higher in low-risk AF patients in sinus rhythm for 6 months after cardioversion compared to a reference group without AF.

Sustained sinus rhythm did not translate into lowered procoagulant activity in our material. These findings support current risk stratification schemes in which stroke risk in AF is considered independent of whether AF is categorised into paroxysmal, persistent and permanent forms [7, 23, 24]. Intriguingly, obtaining sinus rhythm may still be a therapeutic goal in itself, as lower risk of thromboembolism and death has been reported in paroxysmal AF forms [25]. Moreover, the presence of sinus rhythm without antiarrhythmic drugs has been associated with reduced mortality [26, 27]. It is not clear whether the increased stroke risk associated with atrial tachyarrhythmias [28] and rhythm shifts in AF [29–31] is outbalanced by a lower risk during prolonged periods of sinus rhythm [26, 27].

D-dimer has been shown to predict thromboembolic events, and has an additional predictive value to clinical risk scores in patients with AF [10–12]. However, none of the markers were predictive of rhythm outcome after electrical cardioversion in the present AF population. There were no differences in levels of ETP between AF patients maintaining sinus rhythm for 6 months and the reference group. Further studies are warranted to elucidate the relevance of ETP as a marker of hypercoagulability in AF patients.

### Study limitations

The present study was a substudy of the CAPRAF study, and not primarily designed to test the effects of sinus rhythm restoration on markers of thrombin generation or the predictive abilities of these markers in electrical cardioversion of AF, nor was long-term electrocardiogram monitoring conducted as part of this study. Hence we cannot exclude the possibility that some of the AF subjects in sinus rhythm or some of the subjects in the reference group had asymptomatic, paroxysmal AF. Because of small numbers and demographic differences between the reference group and AF patients, our results need confirmation in larger, prospective trials.

## Conclusions

Sustained sinus rhythm after electrical cardioversion of AF did not alter indices of hypercoagulability. In patients with AF and a low CHA_2_DS_2_-VASc score, discontinuation of oral anticoagulation was associated with a rise in D-dimer and F1 + 2 to levels significantly higher than in the reference group. Thus, our data support the current view of sustained hypercoagulability in AF patients despite long-term maintenance of sinus rhythm. Moreover, levels of prothrombotic markers were not associated with rhythm outcome after cardioversion.

## Abbreviations

ACE 1950 study: Akershus Cardiac Examination 1950 study
AF: Atrial fibrillation
CAPRAF study: Candesartan in the prevention of relapsing atrial fibrillation study
CAT assay: calibrated automated thrombogram assay
CHA_2_DS_2_-VASc score: A measure of stroke risk in patients with atrial fibrillation, with scores ranging from 0 to 9 and higher scores indicating greater risk
ETP: Endogenous thrombin potential
F1 + 2: Prothrombin fragment 1 + 2
INR: International normalised ratio
SR: Sinus rhythm

## References

[1] Wolf PA, Dawber TR, Thomas HE, Kannel WB (1978). Epidemiologic assessment of chronic atrial fibrillation and risk of stroke: the Framingham study. Neurology.

[2] Watson T, Shantsila E, Lip GY (2009). Mechanisms of thrombogenesis in atrial fibrillation: Virchow's triad revisited. Lancet.

[3] Choudhury A, Lip GY (2003). Atrial fibrillation and the hypercoagulable state: from basic science to clinical practice. Pathophysiol Haemost Thromb.

[4] Lip GY (1995). Does atrial fibrillation confer a hypercoagulable state?. Lancet.

[5] Jacob K, Talwar S, Copplestone A, Gilbert TJ, Haywood GA (2004). Activation of coagulation occurs after electrical cardioversion in patients with chronic atrial fibrillation despite optimal anticoagulation with warfarin. Int J Cardiol.

[6] Giansante C, Fiotti N, Miccio M, Altamura N, Salvi R, Guarnieri G (2000). Coagulation indicators in patients with paroxysmal atrial fibrillation: effects of electric and pharmacologic cardioversion. Am Heart J.

[7] Kirchhof P, Benussi S, Kotecha D, Ahlsson A, Atar D, Casadei B, Castella M, Diener HC, Heidbuchel H, Hendriks J, Hindricks G, Manolis AS, Oldgren J, Popescu BA, Schotten U, Van Putte B, Vardas P (2016). 2016 ESC guidelines for the management of atrial fibrillation developed in collaboration with EACTS. Eur Heart J.

[8] Spronk HM, De Jong AM, Verheule S, De Boer HC, Maass AH, Lau DH, Rienstra M, van Hunnik A, Kuiper M, Lumeij S, Zeemering S, Linz D, Kamphuisen PW, Ten Cate H, Crijns HJ, Van Gelder IC, van Zonneveld AJ, Schotten U (2017). Hypercoagulability causes atrial fibrosis and promotes atrial fibrillation. Eur Heart J.

[9] Ohara K, Inoue H, Nozawa T, Hirai T, Iwasa A, Okumura K, Lee JD, Shimizu A, Hayano M, Yano K (2008). Accumulation of risk factors enhances the prothrombotic state in atrial fibrillation. Int J Cardiol.

[10] Christersson C, Wallentin L, Andersson U, Alexander JH, Ansell J, De Caterina R, Gersh BJ, Granger CB, Hanna M, Horowitz JD, Huber K, Husted S, Hylek EM, Lopes RD, Siegbahn A (2014). D-dimer and risk of thromboembolic and bleeding events in patients with atrial fibrillation--observations from the ARISTOTLE trial. J Thromb Haemost.

[11] Vene N, Mavri A, Košmelj K, Stegnar M (2003). High D-dimer levels predict cardiovascular events in patients with chronic atrial fibrillation during oral anticoagulant therapy. Thromb Haemost.

[12] Wu N, Chen X, Cai T, Wu L, Xiang Y, Zhang M, Li Y, Song Z, Zhong L (2015). Association of inflammatory and hemostatic markers with stroke and thromboembolic events in atrial fibrillation: a systematic review and meta-analysis. Can J Cardiol.

[13] Tveit A, Grundvold I, Olufsen M, Seljeflot I, Abdelnoor M, Arnesen H, Smith P (2007). Candesartan in the prevention of relapsing atrial fibrillation. Int J Cardiol.

[14] Berge T, Vigen T, Pervez MO, Ihle-Hansen H, Lyngbakken MN, Omland T, Smith P, Steine K, Røsjø H, Tveit A (2015). Heart and brain interactions - the Akershus cardiac examination (ACE) 1950 study design. Scand Cardiovasc J.

[15] Hemker HC, Giesen P, Al Dieri R, Regnault V, de Smedt E, Wagenvoord R, Lecompte T, Béguin S (2003). Calibrated automated thrombin generation measurement in clotting plasma. Pathophysiol Haemost Thromb.

[16] Tveit A, Bollmann A, Seljeflot I, Husser D, Stridh M, Sörnmo L, Arnesen H, Olsson SB, Smith P (2009). Relation between atrial fibrillatory rate and markers of inflammation and haemostasis in persistent human atrial fibrillation. Thromb Haemost.

[17] Munger MA (2011). Use of Angiotensin receptor blockers in cardiovascular protection: current evidence and future directions. PT.

[18] Li-Saw-Hee FL, Blann AD, Gurney D, Lip GY (2001). Plasma von Willebrand factor, fibrinogen and soluble P-selectin levels in paroxysmal, persistent and permanent atrial fibrillation. Effects of cardioversion and return of left atrial function. Eur Heart J.

[19] Marín F, Roldán V, Climent VE, Ibáñez A, García A, Marco P, Sogorb F, Lip GY (2004). Plasma von Willebrand factor, soluble thrombomodulin, and fibrin D-dimer concentrations in acute onset non-rheumatic atrial fibrillation. Heart.

[20] Li-Saw-Hee FL, Blann AD, Lip GY (2000). Effects of fixed low-dose warfarin, aspirin-warfarin combination therapy, and dose-adjusted warfarin on thrombogenesis in chronic atrial fibrillation. Stroke.

[21] Brodin E, Seljeflot I, Arnesen H, Hurlen M, Appelbom H, Hansen JB (2009). Endogenous thrombin potential (ETP) in plasma from patients with AMI during antithrombotic treatment. Thromb Res.

[22] Nozawa T, Inoue H, Iwasa A, Okumura K, Jong-dae L, Shimizu A, Hayano M, Yano K (2004). Effects of anticoagulation intensity on hemostatic markers in patients with non-valvular atrial fibrillation. Circ J.

[23] Friberg L, Hammar N, Rosenqvist M (2010). Stroke in paroxysmal atrial fibrillation: report from the Stockholm cohort of Atrial fibrillation. Eur Heart J.

[24] Disertori M, Franzosi MG, Barlera S, Cosmi F, Quintarelli S, Favero C, Cappellini G, Fabbri G, Maggioni AP, Staszewsky L, Moroni LA, Latini R (2013). Thromboembolic event rate in paroxysmal and persistent atrial fibrillation: data from the GISSI-AF trial. BMC Cardiovasc Disord.

[25] Ganesan AN, Chew DP, Hartshorne T, Selvanayagam JB, Aylward PE, Sanders P, McGavigan AD (2016). The impact of atrial fibrillation type on the risk of thromboembolism, mortality, and bleeding: a systematic review and meta-analysis. Eur Heart J.

[26] Corley SD, Epstein AE, DiMarco JP, Domanski MJ, Geller N, Greene HL, Josephson RA, Kellen JC, Klein RC, Krahn AD, Mickel M, Mitchell LB, Nelson JD, Rosenberg Y, Schron E, Shemanski L, Waldo AL, Wyse DG (2004). Relationships between sinus rhythm, treatment, and survival in the Atrial fibrillation follow-up investigation of rhythm management (AFFIRM) study. Circulation.

[27] Hunter RJ, McCready J, Diab I, Page SP, Finlay M, Richmond L, French A, Earley MJ, Sporton S, Jones M, Joseph JP, Bashir Y, Betts TR, Thomas G, Staniforth A, Lee G, Kistler P, Rajappan K, Chow A, Schilling RJ (2012). Maintenance of sinus rhythm with an ablation strategy in patients with atrial fibrillation is associated with a lower risk of stroke and death. Heart.

[28] Healey JS, Connolly SJ, Gold MR, Israel CW, Van Gelder IC, Capucci A, Lau CP, Fain E, Yang S, Bailleul C, Morillo CA, Carlson M, Themeles E, Kaufman ES, Hohnloser SH (2012). Subclinical atrial fibrillation and the risk of stroke. N Engl J Med.

[29] Boriani G, Botto GL, Padeletti L, Santini M, Capucci A, Gulizia M, Ricci R, Biffi M, De Santo T, Corbucci G, Lip GY (2011). Improving stroke risk stratification using the CHADS2 and CHA2DS2-VASc risk scores in patients with paroxysmal atrial fibrillation by continuous arrhythmia burden monitoring. Stroke.

[30] Botto GL, Padeletti L, Santini M, Capucci A, Gulizia M, Zolezzi F, Favale S, Molon G, Ricci R, Biffi M, Russo G, Vimercati M, Corbucci G, Boriani G (2009). Presence and duration of atrial fibrillation detected by continuous monitoring: crucial implications for the risk of thromboembolic events. J Cardiovasc Electrophysiol.

[31] Glotzer TV, Daoud EG, Wyse DG, Singer DE, Ezekowitz MD, Hilker C, Miller C, Qi D, Ziegler PD (2009). The relationship between daily atrial tachyarrhythmia burden from implantable device diagnostics and stroke risk: the TRENDS study. Circ Arrhythm Electrophysiol.

